# Gut microbiota disorders cause type 2 diabetes mellitus and homeostatic disturbances in gut-related metabolism in Japanese subjects

**DOI:** 10.3164/jcbn.18-101

**Published:** 2019-03-23

**Authors:** Kazunori Adachi, Tomoya Sugiyama, Yoshiharu Yamaguchi, Yasuhiro Tamura, Shinya Izawa, Yasutaka Hijikata, Masahide Ebi, Yasushi Funaki, Naotaka Ogasawara, Chiho Goto, Makoto Sasaki, Kunio Kasugai

**Affiliations:** 1 Department of Gastroenterology, Aichi Medical University School of Medicine, 1-1 Yazakokarimata, Nagakute, Aichi 480-1195, Japan; 2 Department of Health and Nutrition, Faculty of Health and Human Life, Nagoya Bunri University

**Keywords:** gut dysbiosis, short-chain fatty acid, type 2 diabetes, dietary habit, *Bifidobacterium* spp.

## Abstract

Few studies have investigated the host-microbe metabolic axis in people with type 2 diabetes mellitus (T2DM). This study aimed to determine and compare the nutrient intakes and metabolic markers and to elucidate the relationships among these factors in Japanese T2DM patients and control individuals. Fifty-nine Japanese T2DM patients and 59 matched healthy control individuals participated in this study. We examined the differences regarding the participants’ dietary habits, microbiota, and fecal short-chain fatty acids, and analyzed the relationships between the gut microbiota and blood metabolic markers in the T2DM patients and the control subjects. The T2DM patients consumed more carbohydrates, and had lower fecal propionate and butyrate concentrations, larger fecal populations of *Bifidobacterium* spp. and bacteria of the order *Lactobacillales*, and smaller fecal *Bacteroides* spp. populations than the control individuals. In the T2DM patients, the level of *Bifidobacterium* spp. correlated negatively with the carbohydrate intake and the level of bacteria of the order *Lactobacillales* correlated negatively with the protein intake. T2DM patients have gut dysbiosis that may contribute to disease onset and influence its prognosis. Furthermore, homeostatic disturbances in the gut-related metabolism may underlie the pathogenesis of T2DM.

## Introduction

The human gut hosts trillions of microorganisms, and these include over 10^14^ bacteria that belong to 1,000 species.^(1)^ The size of this microbial organ’s genome, which is called the microbiome, exceeds that of the human nuclear genome by 100-fold, and it provides additional biological and metabolic functions that maintain homeostasis within the body.^(2)^

Type 2 diabetes mellitus (T2DM) is a metabolic disorder characterized by hyperglycemia that is caused by deficiencies in insulin production, which are the consequence of the exhaustion of pancreatic beta cell function after insulin resistance has been established.^(3)^ Data from the World Health Organization indicate that about 382 million people worldwide had T2DM in 2013 and that about 592 million people will have T2DM by 2035. In addition, 85% of the premature deaths associated with metabolic syndrome occur in developing countries, and about 80% of these deaths are associated with diabetes.^(4)^ Genetic and environmental factors contribute to the pathogenesis of diabetes, particularly T2DM.^(5–7)^ The findings from recent studies have suggested that the gut microbiota in T2DM patients differs from that in healthy people, and that the gut microbiota is an important environmental factor in diabetes development.^(8)^ Furthermore, compositional changes in particular species of the bacterial genus *Bacteroides* in the intestines of humans and animal models may cause many chronic diseases, including diabetes, obesity, inflammatory bowel disease, cancer, and cardiovascular disease.^(9)^ However, the microbiota of the gut differs according to species, geographic location, age, the gender composition of the population, and dietary habits. Several changes in the gut microbiota were associated with age and sex in healthy Japanese subjects.^(10)^

Short-chain fatty acids (SCFAs), which arise mainly from bacterial fermentation in the gut, can provide energy and they may be involved in lipid and glucose metabolism, which suggests that they may influence metabolic risk factors.^(11)^ We have reported associations between the intestinal microbiota, and, hence, gut SCFAs, and the metabolic markers in and dietary habits of patients with T2DM.^(12)^ However, differences between healthy control individuals and patients with T2DM regarding the associations between fecal SCFAs and metabolism have not yet been described.

This study aimed to determine and compare the nutrient intakes, metabolic markers, including those associated with glucose tolerance, lipid metabolism, and hepatic function, fecal microbiota, and the SCFAs, and to elucidate the relationships among these factors in Japanese T2DM patients and control individuals.

## Methods

This study was conducted according to the World Medical Association Declaration of Helsinki and was approved by the ethics review committee at Aichi Medical University’s School of Medicine in Japan on July 3, 2015 (registration number was 15-019). Written informed consent was obtained from each individual before study enrollment. We recruited Japanese patients who were diagnosed with T2DM using the Japan Diabetes Society’s criteria, and who had glycated hemoglobin (HbA1c) levels of 6.2–7.9% at screening, had received stable doses of medication for at least 1 month, and had stable diabetes for at least 3 months, that is, a change in the HbA1c level of <1.0%. The patients received nutritional guidance from a dietitian, and they were eligible to receive diet therapy. Age- and sex-matched control individuals who did not have histories of diabetes or cancer were recruited to participate in the study. Individuals with a history of gut resection were excluded from the study. Fasting blood and fecal samples were collected and stored at −80°C until they were analyzed. The fecal microbiota was analyzed using the terminal-restriction fragment length polymorphism (T-RFLP) technique. A self-administered short food frequency questionnaire (FFQ) was used to evaluate the subjects’ nutrient intakes.^(13)^

### Fecal DNA extraction

The fecal samples (500 mg) were washed three times with sterile distilled water, suspended in 4 M guanidinium thiocyanate, 100 mM Tris-HCl (pH 9.0), and 40 mM EDTA, and beaten with glass beads using a mini-bead beater (Bio Spec Products Inc., Bartlesville, OK). Thereafter, the DNA was extracted from the bead-treated suspension using benzyl chloride, as described by Zhu *et al.*^(14)^ The DNA extract was then purified using a GFX^TM^ PCR DNA and Gel Band Purification Kit (GE Healthcare, Chicago, IL). The final concentration of each DNA sample was adjusted to 10 ng/µl.

### Terminal-restriction fragment length polymorphism technique

The 16S rDNA amplification and its digestion with restriction enzymes, the size fractionation of the terminal restriction fragments (T-RFs), and the analysis of the T-RFLP data were performed according to the protocol described by Nagashima *et al.*^(15)^ Briefly, a polymerase chain reaction (PCR) was performed using a total fecal DNA (10 ng/µl) sample and an *Escherichia coli*-specific primer pair for 16S rDNA amplification (16f: 5'-TGC CAGCAGCCGCGGTA-3' for *E. coli* positions 516–532 and 1510r: 5'-GGTTACCTTGTTACGACTT-3' for *E. coli* positions 1510–1492). The 5'-ends of the forward primers were labeled with 6'-carboxyfluorescein, which was synthesized by Applied Biosystems Inc. (Foster City, CA). The amplified 16S rDNA genes were purified using a GFX^TM^ PCR DNA and Gel Band Purification Kit (GE Healthcare) and dissolved in 30 µl distilled water. The purified PCR products (2 µl) were digested with *Bsl* I (10 U) at 55°C for 3 h. The lengths of the T-RFs were determined using an ABI Prism^®^ 3130xl genetic analyzer (Applied Biosystems Inc.) in GeneScan mode and standard size markers (MapMarker^®^ X-Rhodamine Labeled 50–1,000 bp; BioVentures, Inc., Murfreesboro, TN). The fragment sizes were estimated using the Local Southern Method sizing algorithm (GeneMapper^®^; Applied Biosystems Inc.).

The T-RFs were divided into 30 operational taxonomic units (OTUs) according to the method described by Nagashima *et al.*^(15)^ Each OTU was quantified as a percentage of the total OTU area, and they were expressed as the peak percent area under the curve (%). Cluster analyses that were based on *Bsl* I T-RFLP patterns, were performed using GeneMaths software (Applied Maths NV, Sint-Martens-Latem, Belgium).

### Fecal short-chain fatty acid analysis

To analyze the SCFAs, feces (0.1 g) were placed in a 2.0 ml tube with zirconia beads and suspended in a 0.1 mM perchloric acid solution with 3% phenol. The samples were heated at 80°C for 15 min, vortexed at 5 m/s for 45 s using a FastPrep 24 homogenizer (MP Biomedicals, Santa Ana, CA), and centrifuged at 15,350 × *g* for 10 min. The supernatant was filtered using a 0.45 µm filter (DISMIC-13HP; Advantec Co., Ltd., Tokyo, Japan). The acetic acid, propionic acid, butyric acid, isobutyric acid, succinic acid, lactic acid, formic acid, valeric acid, and isovaleric acid levels were determined using high-performance liquid chromatography (Prominence; Shimadzu Corporation, Kyoto, Japan) and a post-column reaction detection system, comprising a detector (CDD-10A; Shimadzu Corporation), two columns arranged in tandem (Shim-pack SCR-102(H), 300 mm × 8 mm ID; Shimadzu Corporation), and a guard column (Shim-pack SCR-102(H), 50 mm × 6 mm ID; Shimadzu Corporation). The system included a mobile phase that comprised 5 mM p-toluenesulfonic acid, and a reaction solution that comprised 5 mM p-toluenesulfonic acid, 100 µM EDTA, and 20 mM Bis-Tris. The flow rate was 0.8 ml/min and the oven temperature was 45°C. The detector cell temperature was maintained at 48°C.

### Self-administered short food frequency questionnaire

The FFQ inquired about the subjects’ habitual dietary intakes of 47 foods/recipes during the previous year and their intake frequencies, which were categorized as never or seldom, 1–3 times/month, 1–2 times/week, 3–4 times/week, 5–6 times/week, once/day, twice/day, and >3 times/day. The serving sizes were recorded for staple foods, including rice, noodles, and bread.

### Calculation of nutrient intakes

We computed the average daily consumptions of energy and selected nutrients using the information from the FFQ and a lifestyle questionnaire, which included information about the consumption of alcohol. Based on a regression analysis, selected nutrients were adopted as dependent parameters, and the foods/food groups consumed, intake frequencies, portion sizes in grams, which were based on values from a database or typical/standard values from the literature, and the nutritional content/100 g of the foods/food groups that were listed in composition tables or in model recipes, were considered to be independent variables.^(16–18)^

### Statistical analyses

All of the statistical analyses were performed using JMP statistical software, ver. 11.2.1 for Windows (SAS Institute Inc., Cary, NC). The normally distributed data are expressed as the means and the standard deviations, and the data with skewed distributions are presented as the medians (interquartile ranges). Spearman’s correlation coefficient was used to determine associations between the abundance of the fecal bacteria and the SCFAs, food intakes, and the clinical parameters. A value of *p*<0.05 was considered statistically significant.

## Results

### Patients’ characteristics and food intakes

 Fifty-nine patients with T2DM, comprising 34 men and 25 women whose ages ranged from 57.5 years to 69 years (median 64 years), and 59 control individuals, comprising 34 men and 25 women whose ages ranged from 59 years to 69 years (median 62 years), were enrolled to participate in this study. Table 1 presents the characteristics of the patients with T2DM and the control individuals. The groups did not differ significantly regarding the average body mass index (BMI), and the high-density lipoprotein cholesterol (HDL-C), triglyceride (TG), free fatty acid (FFA), total cholesterol (TC), low-density lipoprotein cholesterol (LDL-C), γ-glutamyl transferase (γ-GTP), and aspartate aminotransferase (AST) levels. The T2DM patients had higher fasting blood glucose (FBG), HbA1c, insulin, and alanine aminotransferase (ALT) levels, and homeostasis model of insulin resistance (HOMA-IR) values, than the control subjects. Based on the data from the FFQ, the mean total energy intakes were similar in the T2DM patients (1,692 ± 380 kcal/day) and the control subjects (1,705 ± 345 kcal/day). The ratio of fat intake and protein intake to total energy intake in the T2DM patients and in the control subjects was 23.2 ± 5.3% vs 24.7 ± 6.3%, and 13.2 ± 2.2% vs 12.7 ± 1.7%, respectively; there were not significantly different between the two groups. The mean carbohydrate intake as a proportion of the total energy intake was significantly higher in the T2DM patients than that in the control subjects (57.5 ± 5.2% vs 55.4 ± 5.5%, *p*<0.05). The number of the participants who consumed yogurt at least once a week was significantly lower in the T2DM patient group (*n* = 27) than that in the control group (*n* = 41) (*p*<0.01).

### Cluster analysis

**Fig. 1:**
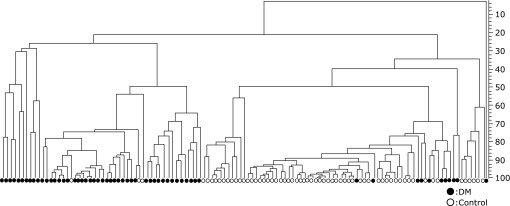
Dendrogram of the fecal bacteria structure in the T2DM patients and control subjects. T-RFLP patterns by *BslI* digestions were analyzed using the software GeneMaths (Applied Maths), and the Person similarity coefficient analys and unweighted pair-group method with arithmetic means were used to establish the type of dendrogram. Open circle, control individuals; closed sircle, T2DM patients.

Figure 1 showed the dendrogram of the fecal bacteria structure in the T2DM patients and the control individuals. Two separate clusters were evident that corresponded to the T2DM patients and the control subjects.

### Terminal-restriction fragment length polymorphism analysis of the fecal samples

Table 2 shows the population of total sequences and the detection rate in fecal bacteria of the T2DM patients and the control subjects. The population of fecal bacteria for *Bifidobacterium* spp. (25.7% vs 5.5%, *p*<0.01) and for order *Lactobacillales* (17.3% vs 1.6%, *p*<0.01) were significantly higher in the fecal samples from the T2DM patients than those in the fecal samples from the control group. The population of fecal bacteria for *Bacteroides* spp. was significantly lower in the T2DM patient group than that in the control group (12.4% vs 50.6%, *p*<0.01). The population of fecal bacteria for fecal *Clostridium* cluster IV, *Clostridium* cluster XI, *Clostridium* subcluster XIVa, *Clostridium* cluster XVIII , and *Prevotella* spp. were similar in both groups. In the T2DM patient group, the *Clostridium* cluster IV (83.1% vs 96.6%, *p*<0.05), *Clostridium* cluster XI (32.2% vs 54.2%, *p*<0.05), and *Clostridium* cluster XVIII (44.1% vs 86.4%, *p*<0.01) detection rates were significantly lower than those in the control subjects.

### Fecal short-chain fatty acid and pH levels

The total SCFA levels and the fecal pH levels were similar in the two groups (Table 3). The T2DM patients had lower fecal propionate (1.32 mg/g vs 1.81 mg/g, *p*<0.05) and butyrate (1.03 mg/g vs 1.48 mg/g, *p*<0.01) levels and a higher fecal succinate level (0.14 mg/g vs 0.07 mg/g, *p*<0.01) than those in the control subjects. The valerate detection rate was lower in the T2DM patients than that in the control subjects (74.6% vs 94.8%, *p*<0.01). The succinate detection rate was higher in the T2DM patients than that in the control subjects (59.3% vs 25.7%, *p*<0.01).

### Relationships among the fecal microbiota, the short-chain fatty acids, the food intakes, and metabolism

Table 4 shows the relationships among the fecal microbiota, the SCFAs, the food intakes, and metabolism in the T2DM patients and the control subjects. The level of fecal *Bifidobacterium* spp. correlated negatively with the carbohydrate intake in the T2DM patients (*r* = −0.420, *p* = 0.001), but not in the control subjects. In the T2DM patients, the level of fecal bacteria of the order *Lactobacillales* correlated negatively with the protein intake (*r* = −0.275, *p* = 0.035) and the TC level (*r* = −0.317, *p* = 0.016), but not in the control subjects. The level of fecal *Bacteroides* spp. correlated negatively with the FBG level in the T2DM patients (*r* = −0.265, *p* = 0.043), but not in the control subjects. In the T2DM patients, the level of fecal *Clostridium* cluster IV correlated positively with the carbohydrate intake (*r* = 0.266, *p* = 0.042) and negatively with the fat intake (*r* = –0.261, *p* = 0.046), and it correlated negatively with the TC level in the control subjects (*r* = −0.279, *p* = 0.032). The level of fecal *Clostridium* cluster XI correlated positively with the fat intake (*r* = 0.301, *p* = 0.021), protein intake (*r* = 0.362, *p* = 0.005), and the HDL-C level (*r* = 0.277, *p* = 0.034) in the T2DM patients, and in the control subjects, the *Clostridium* cluster XI level correlated positively with the insulin level (*r* = 0.408, *p* = 0.001), HOMA-IR value (*r* = 0.413, *p* = 0.001), and the FBG level (*r* = 0.270, *p* = 0.038), and it correlated negatively with the FFA level (*r* = −0.281, *p* = 0.031). In the T2DM patients, the level of fecal *Clostridium* cluster XIVa correlated positively with the TC level (*r* = 0.321, *p* = 0.014), and in the control subjects, it correlated negatively with the total energy intake (*r* = −0.264, *p* = 0.043). The level of fecal *Clostridium* cluster XVIII correlated negatively with the HOMA-IR value in the T2DM patients (*r* = −0.300, *p* = 0.022), and in the control subjects, it correlated negatively with the LDL-C (*r* = −0.303, *p* = 0.020), FFA (*r* = −0.340, *p* = 0.008), and TC (*r* = −0.325, *p* = 0.012) levels. The level of fecal *Prevotella* spp. correlated negatively with the HbA1c level in the T2DM patients (*r* = −0.271, *p* = 0.038) and with the FFA level in the control subjects (*r* = −0.257, *p* = 0.050). In the T2DM patients, the level of other fecal bacteria correlated positively with the ALT (*r* = 0.331, *p* = 0.011), AST (*r* = 0.258, *p* = 0.049), and γ-GTP (*r* = 0.300, *p* = 0.021) levels, correlations that were not apparent in the control subjects.

The total SCFA level in the feces correlated negatively with the protein intake in the T2DM patients (*r* = −0.275, *p* = 0.038), a correlation that was not apparent in the control subjects. The fecal acetate level correlated negatively with the protein intake (*r* = −0.285, *p* = 0.032), insulin level (*r* = −0.301, *p* = 0.024), and the HOMA-IR value (*r* = −0.284, *p* = 0.034), and it correlated positively with the γ-GTP level (*r* = 0.288, *p* = 0.030) in the T2DM patients, correlations that were not apparent in the control subjects. In the T2DM patients, the fecal propionate level correlated negatively with the insulin level (*r* = −0.269, *p* = 0.045) and the HOMA-IR value (*r* = −0.282, *p* = 0.035), and it correlated positively with the γ-GTP (*r* = 0.416, *p* = 0.001) and TG (*r* = 0.308, *p* = 0.037) levels. In the control subjects, the fecal propionate level correlated negatively with the insulin level (*r* = −0.284, *p* = 0.032) and the HOMA-IR value (*r* = −0.278, *p* = 0.036) and it correlated positively with the γ-GTP (*r* = 0.405, *p* = 0.002) and ALT (*r* = 0.292, *p* = 0.028) levels. The fecal butyrate level correlated positively with the HDL-C level in the T2DM patients (*r* = 0.303, *p* = 0.020), a correlation that was not apparent in the control subjects. The fecal valerate level correlated negatively with the ALT level in the T2DM patients (*r* = −0.298, *p* = 0.022) and it correlated positively with the γ-GTP level in the control subjects (*r* = 0.370, *p* = 0.004). The fecal succinate level correlated positively with the TC (*r* = 0.275, *p* = 0.036) and FBG (*r* = 0.304, *p* = 0.020) levels, and with the total energy intake (*r* = 0.316, *p* = 0.016) in the control subjects, correlations that were not apparent in the T2DM patients.

### Relationships between medication and the fecal microbiota

We investigated differences in the gut microbiota in the presence and absence of metformin and α-glucosidase inhibitor (α-GIs). The fecal level of *Clostridium* cluster XI was significantly lower in the patients who were treated with metformin than that in the patients who were not treated with metformin (0.9 ± 3.6% vs 1.8 ± 3.2%, *p* = 0.04). The fecal level of the other bacteria was significantly higher in the patients who were treated with metformin than that in the patients who were not treated with metformin (18.5 ± 13.4% vs 9.7 ± 5.7%, *p* = 0.009). In the patients who were treated with α-GIs, the fecal levels of *Bifidobacterium* spp. (32.2 ± 10.3% vs 17.6 ± 13.7%, *p*<0.001) and bacteria of the order *Lactobacillales* (27.1 ± 14.8% vs 10.3 ± 10.3%, *p*<0.001) were significantly higher and the fecal levels of *Clostridium* cluster IV (3.8 ± 4.9% vs 9.8 ± 8.14%, *p* = 0.008), *Clostridium* subcluster XIVa (11.2 ± 8.8% vs 26.8 ± 11.6%, *p*<0.001), and other bacteria (9.1 ± 5.9% vs 14.8 ± 11.0%, *p* = 0.017) were significantly lower than those in the patients those who were not treated with α-GIs.

## Discussion

The main finding from this study was that the Japanese patients with T2DM had gut dysbiosis. Previous investigators have described gut dysbiosis in patients with T2DM of different races,^(19–23)^ and this is the second study to compare the gut microbiota in Japanese patients with T2DM with that in age- and sex-matched healthy control individuals. The Japanese diet is very different from westernized diets, and the gut microbiota within Japanese patients with T2DM might differ from that in westernized individuals; therefore, it is important to investigate the differences between Japanese T2DM patients and healthy subjects in relation to the gut microbiota. The second major finding from this study comprised the differences between the T2DM patients and the healthy subjects regarding the relationships between the enteral environment, food intake, and the metabolic blood markers, and this is the first time that this has been described. 

In the present study, the level of bacteria of the order *Lactobacillales* was significantly higher in the patients with T2DM than that in the control subjects. This observation concurs with findings from Japanese^(19)^ and westernized^(20–23)^ individuals. The reason underlying the higher level of bacteria of the order *Lactobacillales* is unclear, but they are innate bacteria, because compared with the control subjects, significantly fewer T2DM patients consumed yogurt. In addition, and in contrast to previous studies,^(19,20,22)^ the patients with T2DM in the present study were not obese, and the BMI did not differ between the study groups. Therefore, the higher level of bacteria of the order *Lactobacillales* might play an important role in the pathogenesis of T2DM, and further studies are necessary to investigate the roles these bacteria play in the development of T2DM.

The patients with T2DM had significantly lower levels of *Bacteroides* spp. than those in the control subjects, a finding that conflicts with the findings from studies involving Chinese^(24)^ and Japanese^(19)^ patients with T2DM. Qin *et al.*^(24)^ reported that compared with control subjects, the fecal level of *Bacteroides* spp. was significantly higher in patients with diabetes, while Sato *et al.*^(19)^ reported that there was no difference between patients with diabetes and control subjects regarding the level of *Bacteroides* spp. The reason for these differences is unclear, but dietary habits or the BMI might affect the levels of *Bacteroides* spp. The BMI of the patients with T2DM in this study was lower than that of the patients with T2DM in the study conducted by Sato *et al.*^(19)^ The results from a recent study showed that *Bacteroides* and other commensal bacterial species affected the intestinal mucus and the glycocalyx, which may influence intestinal permeability.^(25)^ Further studies are needed to investigate the roles of *Bacteroides* spp. in T2DM.

In this study, the level of *Bifidobacterium* spp. was significantly higher in the patients with T2DM than that in the control subjects, which differs from the findings from previous studies. Sedighi *et al.*^(23)^ reported that compared with control subjects, the level of *Bifidobacterium* spp. was significantly lower in patients with T2DM. Differences in relation to the analyses of the microbiota might explain the dissimilar findings. Sato *et al.*^(19)^ reported that the level of *Bifidobacterium* did not differ between control subjects and patients with T2DM. α-GIs affect the gastrointestinal system, and they increase the levels of *Bifidobacterium* spp. and *Lactobacillus* spp.^(19,26,27)^ In the present study, the levels of *Bifidobacterium* spp. and bacteria of the order *Lactobacillales* were higher in the patients with T2DM who were treated with α-GIs. The difference between the findings from this study and those from the study performed by Sato *et al.*^(19)^ regarding the level of bacteria of the order *Lactobacillales* might be associated with the number of patients treated with α-GIs.

Sato *et al.*^(19)^ reported that the total SCFA concentration in patients with T2DM was significantly lower than that in control subjects, which disagrees with the findings from the present study. Complex carbohydrates, such as those in dietary fiber, are metabolized into oligosaccharides and monosaccharides by the colonic microbiota, and they undergo fermentation to form SCFAs.^(28)^ Therefore, differences in relation to the uptake of carbohydrates by the study subjects might explain these divergent findings.

Acetate, propionate, and butyrate comprise 90–95% of the SCFAs present in the colon,^(29)^ and the intraluminal SCFA concentrations consist of 60% acetate, 25% propionate, and 15% butyrate.^(30)^ SCFAs contribute approximately 5–10% of human energy requirements.^(31)^ In addition, SCFAs stimulate glucagon-like peptide-1 secretion via a G-protein-coupled receptor.^(32)^ In this study, the acetate and propionate levels correlated negatively with the level of insulin in the blood and the HOMA-IR value. Therefore, a decrease in the fecal propionate level may lead to glucose intolerance in T2DM patients.

In the present study, associations among the fecal microbiota, organic acids, and a variety of clinical parameters were identified. However, the associations in the T2DM patients and those in the control subjects were markedly different. This is supported by Inoue’s study which presented the difference of functional profile of microbiota between Japanese control subjects and T2DM patients from 16S rRNA metagenomic data.^(33)^ In addition, many associations between food intakes and the clinical parameters were identified in the patients with T2DM, but they were not apparent in the control subjects. Carbohydrate and protein intake were associated with fecal abundance of *Bifidobacterium* spp. and order *Lactobacillales* in T2DM patients but not in control subjects. The reasons for these differences are unclear, but they could suggest that the homeostasis among the gut microbiota and organs is disrupted in T2DM patients. Cani *et al.*^(34)^ reported that a high-fat diet triggers gut dysbiosis and subsequently causes insulin resistance via a lipopolysaccharide-dependent mechanism. High-fat food increased the translocation of live gram-negative bacteria through the intestinal mucosa and into the blood and mesenteric adipose tissue in mice, which was, in turn, linked to low-grade inflammation.^(35)^ Sato *et al.*^(19)^ reported that the level of interleukin-6, which was enhanced by lipoteichoic acid in a variety of cell types in other studies,^(36)^ was higher in patients with T2DM, and they detected gut bacteria in the blood of these patients. These changes might contribute to the disruption of organ homeostasis. Further studies are needed to investigate the mechanism underlying the relationships between the enteral environment and the clinical parameters.

The present study has some limitations. First, this study included a relatively small number of patients, and it was conducted at a single center. Further large-scale multicenter studies are needed to clarify the role of the gut microbiota in T2DM. Secondly, this study did not consider age-related changes in the gut microbiota. Age-related changes in the gut microbiota have been described in individuals aged >70 years, which included an increase in the level of *Bacteroides* spp.^(37)^ In this study, there was no relationship between age and the gut microbiota (data not shown), but this may have been a consequence of the small number of patients reducing the study’s analytical power. Further studies may be necessary to clarify the influence of age on the gut microbiota, and many more control subjects and T2DM patients will be required to determine the differences in gut dysbiosis with age.

## Conclusion

Patients with T2DM have gut dysbiosis that may contribute to the onset of the disease and affect its prognosis. Furthermore, disturbances in the homeostasis of gut-related metabolism may underlie the pathogenesis of T2DM.

## Figures and Tables

**Table 1:** Characteristics of the study subject

	Type 2 diabetic patients (*n* = 59)	Control subjects (*n* = 59)
SEX, *n*		
Male	34	34
Female	25	25
Age	64.0 (57.5–69.0)	62.0 (59.0–69.0)
30–39, M : F	2 : 0	2 : 0
40–49, M : F	0 : 1	0 : 1
50–59, M : F	10 : 5	10 : 5
60–69, M : F	12 : 15	12 : 15
70–79, M : F	10 : 4	10 : 4
Body mass index, kg/m^2^	23.0 (20.4–25.6)	22.7 (20.8–24.3)
FBG, mg/dl	144.7 ± 27.1**	96.3 ± 10.3
HbA1c, %	7.1 (6.8–7.6)**	5.5 (5.3–5.7)
Insulin, µU/ml	7.2 (4.9–11.1)*	5.3 (3.1–7.5)
HOMA-IR	2.7 (1.7––4.5)**	1.2 (0.7–1.7)
TC, mg/dl	208.6 ± 32.0	211.4 ± 35.0
HDL, mg/dl	57.7 ± 16.9	60.8 ± 15.9
LDL, mg/dl	124.7 ± 29.0	131.2 ± 28.9
TG, mg/dl	123.3 ± 47.7	110.7 ± 61.8
FFA, Eq/L	645.4 ± 436.3	500.5 ± 248.0
AST, U/L	21.0 (20.0–24.5)	21.0 (19–24.5)
ALT, U/L	21.0 (17.0–28.0)*	18.0 (14.0–23.0)
γ-GTP, U/L	22.0 (17.5–32.0)	24.0 (16.0–38.5)
Medication for diabetes, *n*	56	0
No medication	3	59
Insulin only or with oral therapy	11	0
Oral therapy only	45	0
SU	34	0
Metformin	17	0
α-GI	27	0
Thiazolidine	15	0
Medication for other diseases, *n*	45	18
No medication	14	41
Antihypertensive drugs	28	9
Lipid-lowering drugs	27	10
Total energy intake, kcal/day	1,692 ± 380	1,705 ± 345
Carbohydrate intake, %	57.5 ± 5.2*	55.4 ± 5.5
Fat intake, %	23.2 ± 5.3	24.7 ± 6.3
Protein intake, %	13.2 ± 2.2	12.7 ± 1.7
Participants taking yogurt at least once a week, *n*	27**	41

The data presented are the means and the SD (coefficient of variation) or the medians (interquartile ranges), unless otherwise indicated. *p<0.05 vs control, **p<0.01 vs control. M, male; F, female; FBG, fasting blood glucose; HbA1c, hemoglobin A1c; HOMA-IR, homeostasis model assessment for insulin resistance; TC, total cholesterol; HDL, high-density lipoprotein cholesterol; LDL, low-density lipoprotein cholesterol; TG, triglyceride; FFA, free fatty acid; AST, aspartate aminotransferase; ALT, alanine aminotransferase; γ-GTP, γ-glutamyl transferase; SU, sulfonylurea; α-GI, α-glucosidase inhibitor.

**Table 2:** Terminal restriction fragment length polymorphism analysis of the feces

	Type 2 diabetic patients			Control subjects	
	Population, % of total sequences	Detection rate, %		Population, % of total sequences	Detection rate, %
*Bifidobacterium* spp.	25.7 (12.4–38.5)**	94.9		5.5 (1.5–12.0)	88.1
Order *Lactobacillales*	17.3 (5.7–29.3)**	89.8		1.6 (0.8–3.5)	91.5
*Bacteroides* spp.	12.4 (4.8–15.8)**	98.3		50.6 (42.2–55.1)	100
*Clostridium* cluster IV	3.4 (1.3–13.4)	83.1*		6.4 (3.4–8.7)	96.6
*Clostridium* cluster XI	0.0 (0.0–1.5)	32.2*		0.4 (0–1.2)	54.2
*Clostridium *subcluster XIVa	17.0 (8.0–29.0)	98.3		15.3 (11.2–19.3)	100
*Clostridium* cluster XVIII	0.0 (0.0–4.9)	44.1**		1.1 (0–2.4)	86.4
*Prevotella* spp.	0.0 (0.0–0.0)	11.9		0.0 (0.0–0.0)	22.0
Others	9.2 (6.4–14.8)**	94.9		6.2 (4.7–8.6)	100

The data presented are the medians (interquartile ranges), unless otherwise indicated. *p<0.05 vs control, **p<0.01 vs control.

**Table 3:** Fecal short-chain fatty acid and the pH levels

	Type 2 diabetic patients			Control subjects	
	Fecal SCFAs (mg/g) and pH	Detection rate, %		Fecal SCFAs (mg/g) and pH	Detection rate, %
Total SCFAs	7.05 (4.85–9.53)	100		8.15 (6.44–9.81)	100
Acetate	3.71 (2.53–4.73)	96.6		4.31 (2.67–5.80)	100
Propionate	1.32 (0.83–3.72)*	96.6		1.81 (1.26–2.26)	96.6
Butyrate	1.03 (0.41–1.54)**	86.4		1.51 (0.93–1.98)	100
Valerate	0.61 (0.06–0.99)	74.6**		0.40 (0.24–0.72)	94.8
Succinate	0.14 (0.00–0.11)**	59.3**		0.07 (0.00–0.06)	25.7
Formate	0.00 (0.00–0.00)*	6.8		0.00 (0.00–0.00)	0
Lactate	0.17 (0.00–0.00)	15.3		0.00 (0.00–0.00)	5.1
pH	6.74 ± 0.75	100		6.85 ± 0.85	100

The data presented are the medians (interquartile ranges), unless otherwise indicated. *p<0.05 vs control, **p<0.01 vs control. SCFA, short-chain fatty acid.

**Table 4:** Correlations among the fecal bacteria, organic acids, food intake and the clinical parameters in type 2 diabetic patients and the control subjects

	Type 2 diabetic patients				Control subjects		
	Clinical parameters	*r*	*p*		Clinical parameters	*r*	*p*
*Bifidobacterium* spp.	Carbohydrate intake	–0.420	0.001		None		
Order *Lactobacillales*	Protein intake	–0.275	0.035		None		
	TC	–0.317	0.016				
*Bacteroides* spp.	FBG	–0.265	0.043		None		
*Clostridium* cluster IV	Carbohydrate intake	0.266	0.042		TC	–0.279	0.032
	Fat intake	–0.261	0.046				
*Clostridium* cluster XI	Fat intake	0.301	0.021		Insulin	0.408	0.001
	Protein intake	0.362	0.005		HOMA-IR	0.413	0.001
	HDL	0.277	0.034		FFA	–0.281	0.031
					FBG	0.270	0.038
*Clostridium* subcluster XIVa	TC	0.321	0.014		Total energy intake	–0.264	0.043
*Clostridium* cluster XVIII	HOMA-IR	–0.300	0.022		LDL	–0.303	0.020
					FFA	–0.340	0.008
					TC	–0.325	0.012
*Prevotella* spp.	HbA1c	–0.271	0.038		FFA	–0.257	0.050
Others	ALT	0.331	0.011		None		
	AST	0.258	0.049				
	γ-GTP	0.300	0.021				
Total SCFA	Protein intake	–0.275	0.038		None		
Acetate	Insulin	–0.301	0.024		None		
	HOMA-IR	–0.284	0.034				
	γ-GTP	0.288	0.030				
	Protein intake	–0.285	0.032				
Propionate	Insulin	–0.269	0.045		Insulin	–0.284	0.032
	HOMA-IR	–0.282	0.035		HOMA-IR	–0.278	0.036
	γ-GTP	0.416	0.001		γ-GTP	0.405	0.002
	TG	0.308	0.037		ALT	0.292	0.028
Butyrate	HDL	0.303	0.020		None		
Valerate	ALT	–0.298	0.022		γ-GTP	0.370	0.004
Succinate	None				TC	0.275	0.036
					FBG	0.304	0.020
					Total energy intake	0.316	0.016

Only the statistically-significant items are presented. TC, total cholesterol; FBG, fasting blood glucose; HDL-C, high-density lipoprotein cholesterol; HOMA-IR, homeostasis model assessment for insulin resistance; FFA, free fatty acid; LDL, low-density lipoprotein; HbA1c, hemoglobin A1c; TG, triglyceride; ALT, alanine aminotransferase; AST, aspartate aminotransferase; γ-GTP, γ-glutamyl transferase; SCFA, short chain fatty acid.

## Abbreviations

ALT: alanine aminotransferase
AST: aspartate aminotransferase
FBG: fasting blood glucose
FFA: free fatty acid
FFQ: food frequency questionnaire
γ-GTP: γ-glutamyl transferase
HbA1c: glycated hemoglobin
HDL-C: high-density lipoprotein cholesterol
HOMA-IR: homeostasis model of insulin resistance
LDL-C: low-density lipoprotein cholesterol
OTU: operational taxonomic units
SCFA: short-chain fatty acid
T2DM: type 2 diabetes mellitus
TC: total cholesterol
TG: triglyceride
T-RF: terminal-restriction fragment
T-RFLP: terminal-restriction fragment length polymorphism
